# Fruit and Vegetable Consumption and Potential Moderators Associated with All-Cause Mortality in a Representative Sample of Spanish Older Adults

**DOI:** 10.3390/nu11081794

**Published:** 2019-08-02

**Authors:** Beatriz Olaya, Cecilia A. Essau, Maria Victoria Moneta, Elvira Lara, Marta Miret, Natalia Martín-María, Darío Moreno-Agostino, José Luis Ayuso-Mateos, Adel S. Abduljabbar, Josep Maria Haro

**Affiliations:** 1 Research, Innovation and Teaching Unit, Parc Sanitari Sant Joan de Déu, Carrer Doctor Pujadas 42, 08830 Sant Boi de Llobregat, Spain; 2 Instituto de Salud Carlos III, Centro de Investigación Biomédica en Red de Salud Mental (CIBERSAM), Calle Monforte de Lemos 3–5, 28029 Madrid, Spain; 3 Department of Psychology, University of Roehampton, Whitelands College, London SW15 4JD, UK; 4 Department of Psychiatry, Instituto de Investigación Sanitaria, Hospital Universitario de La Princesa (IIS-Princesa), Calle de Diego de León 62, 28006 Madrid, Spain; 5 Department of Psychiatry, Universidad Autónoma de Madrid, Madrid, Calle Arzobispo Morcillo 4, 28029 Madrid, Spain; 6 Psychology Deparment, King Saud University, Riyadh 11451, Saudi Arabia

**Keywords:** survival, fruit and vegetable consumption, interaction, older adults, multimorbidity

## Abstract

This study sought to determine the association between levels of fruit and vegetable consumption and time to death, and to explore potential moderators. We analyzed a nationally-representative sample of 1699 older adults aged 65+ who were followed up for a period of 6 years. Participants were classified into low (≤3 servings day), medium (4), or high (≥5) consumption using tertiles. Unadjusted and adjusted cox proportional hazard regression models (by age, gender, cohabiting, education, multimorbidity, smoking, physical activity, alcohol consumption, and obesity) were calculated. The majority of participants (65.7%) did not meet the recommendation of five servings per day. High fruit and vegetable intake increased by 27% the probability of surviving among older adults with two chronic conditions, compared to those who consumed ≤3 servings per day (HR = 0.38, 95%CI = 0.21–0.69). However, this beneficial effect was not found for people with none, one chronic condition or three or more, indicating that this protective effect might not be sufficient for more severe cases of multimorbidity. Given a common co-occurrence of two non-communicable diseases in the elderly and the low frequency of fruit and vegetable consumption in this population, interventions to promote consuming five or more servings per day could have a significant positive impact on reducing mortality.

## 1. Introduction

Fruit and vegetable consumption has consistently been associated with beneficial effects on health [1,2]. Several meta-analyses have indicated a reduced risk of non-communicable diseases such as cancer [3], stroke [4], diabetes [5], hypertension [6], and heart diseases [3]. According to the WHO, 16 million disability-adjusted life-years (DALYs) and 1.7 million deaths worldwide are attributable to low fruit and vegetable consumption [7]. High fruit and vegetable intake has been associated with a reduced risk of all-cause mortality [8,9] due to cardiovascular diseases (CVD) [10], but also to other non-cardiovascular diseases [11], such as cancer [12], although findings are inconsistent [13]. Many public health guidelines recommend a daily intake of a minimum of five servings per day of fruit and vegetable, although these recommendations vary across regions. For example, the Eurodiet core report [14], the World Cancer Research Fund [15], and the WHO/FAO [16] recommend at least 400 g/day, 600 g/day in Denmark [17], and 640/800 g/day in the USA [3]. 

Older adults have unique nutritional needs that might require special adaptations of the nutritional clinical guidelines and public health policies addressed to this population [18]. Chronic diseases, multimorbidity, and geriatric conditions such as polypharmacy, mobility-difficulties, and oral problems, are common in older adults and might be associated with malnutrition [19]. The majority of studies of the health benefits of fruit and vegetable consumption have traditionally focused on children, adolescents, or young adults, but few have included older adults [18]. These few studies seem to suggest that fruit and vegetable intake can prevent the onset of depression [20], cognitive decline [21], disability [22], and frailty [23], and can decrease the risk of disease-specific and all-cause mortality in this population [18]. Moreover, the potential benefit of fruit and vegetable intake in reducing the risk of mortality in older adults might depend on various circumstances. For example, a study conducted in several population-based cohorts from Eastern Europe [24] reported that fruit and vegetable consumption was strongly and inversely associated with risk of total and CVD mortality among smokers, compared with non-smokers. 

The present study sought to determine the association between different levels of fruit and vegetable consumption and risk of all-cause mortality in a representative sample of Spanish community-dwelling older adults and to determine potential moderators of this association, including multimorbidity and other lifestyle factors such as smoking, alcohol consumption, and physical activity. 

## 2. Methods

### Study Sample

This study used data from the “Edad con Salud”, a longitudinal household survey of the non-institutionalized adult population in Spain. The first wave took place between 2011 and 2012 and was part of the Collaborative Research on Ageing in Europe (COURAGE) study [25]. The participants were re-evaluated twice, between 2014 and 2015, and in 2018. A stratified multistage clustered design was used, in which strata included Autonomous Communities except Ceuta and Melilla. People aged 50+ were oversampled. The Spanish Statistical Office provided a list of households, and individuals were randomly selected from within the household by the interviewer. A total of 4753 persons participated at baseline with a final response rate of 69.9%. 

Interviews were conducted face to face by trained lay interviewers using Computer-Assisted Personal Interviewing (CAPI) at respondents’ homes. Participants answered a questionnaire adapted from the Study on global AGEing and adult health [26] (SAGE) which was translated from English into Spanish using World Health Organization translation guidelines for assessment instruments [27]. Quality control procedures were implemented during the fieldwork [28]. At the beginning of the interview, the interviewer judged whether the selected person had cognitive limitations that would prevent correct understanding of the survey questions. In such cases, a proxy respondent answered a short version of the interview. Proxy interviews were excluded from the present analysis (*n* = 170). We focused on people aged 65 or older with complete data in all the covariates at baseline, resulting in a final *n* of 1699. Ethical approval was obtained from the Clinical Investigation Ethics Committee, Parc Sanitari Sant Joan de Déu, Barcelona (PIC-12-11; PIC-71-12) and from the Clinical Investigation Ethics Committee, Hospital Universitario la Princesa, Madrid (PI-364; 2399). Informed consent was obtained from each participant.

## 3. Measures

### 3.1. Fruit and Vegetable Consumption

Participants were asked the following questions: “*How many servings of fruit do you eat on a typical day?”* and *“How many servings of vegetables do you eat on a typical day?”*. Respondents were shown a card indicating with pictures and in written explanation what was considered a serving of fruit and vegetables, according to the WHO recommendations [7]. One standard serving (portion) included 80 g, translated into different units of cups depending on the type of fruit and vegetable and standard cup measures available in the country. For example, a piece of banana or apple was considered as one serving. Tubers (such as potatoes) were not included. The total number of fruit and vegetables was added up (ranging from 0 to 18), and a categorical variable was created to indicate the level of consumption using tertiles: low (≤3 servings day), medium (4 servings day), and high (≥5 servings day).

### 3.2. Other Covariates

Socio-demographic information at baseline included age, gender, educational level (no education/primary school, secondary school, and high school/university studies), and current marital status (never married, widowed, separated or divorced, recorded as “not cohabiting”, and married or cohabiting with someone, recorded as “cohabiting”). A binary variable (*ever smoked*) was created including those who had never smoked and those who were current smokers or ex-smokers (including smoking tobacco or using smokeless tobacco). Level of physical activity was evaluated with the Global Physical Activity Questionnaire [29], and participants were classified into high, medium, and low levels [30]. Respondents were asked if they had ever consumed alcohol, the number of days, and standard drinks on average. They were classified as lifetime abstainers (never consumed alcohol), occasional drinkers (did not consume alcohol in the last 30 days or in the last 7 days), non-heavy drinkers (did consume alcohol in the last 30 days and in the last 7 days), infrequent heavy drinkers (did consume alcohol 1–2 days per week, with five or more standard drinks in the last 7 days for men and four or more for women), and frequent heavy drinkers (did consume alcohol 3 or more days per week with five or more standard drinks in the last 7 days for men and four or more for women). Due to the low frequency of heavy drinkers in our sample, non-heavy drinkers, infrequent, and frequent heavy-drinkers were merged into the single category of “frequent drinker”.

A combined method, consisting of self-reported physician’s diagnosis and/or symptom-based algorithms [31], was used to assess the following medical conditions: arthritis, asthma, chronic obstructive pulmonary disease (COPD), angina pectoris, stroke, hypertension, and diabetes. For diabetes, only a self-reported diagnosis was considered. The presence of hypertension was based on self-reported diagnosis or presence of systolic blood pressure ≥ 140 mmHg or diastolic blood pressure ≥ 90 mmHg measured at the time of the interview [32,33]. The number of chronic conditions (CC) was calculated (none or one, 2, and 3 or more CC). Interviewers measured participants’ height and weight using a stadiometer and a routinely calibrated electronic weighing scale, respectively. Body Mass Index (BMI) was calculated as weight (in kilograms) divided by the square of height (in meters). A BMI of 30 or higher was used as cut-off point for obesity [34]. 

### 3.3. Mortality 

The National Death Index, a civil registry for all Spanish residents, was used to ascertain the vital status and date of death for all participants from 25 July 2011 to March 2018. Vital status was also updated during household visits in the follow-up assessment by asking respondents’ relatives. A final update was conducted on the 31 October 2018 by once again consulting the National Death Index. Twelve participants that appeared as deceased had no information about their date of death. Thus, we estimated their date of death as occurring at the mid-point between the date of interview at baseline and 31 October 2018.

### 3.4. Statistical Analysis

Unweighted frequencies, weighted proportions, and means were used for descriptive analyses. Distinct levels of fruit and vegetable consumption were compared using the Rao-Scott chi-squared test statistic (which adjusts for complex sample design) [35] for categorical variables and one-way ANOVA test for continuous variables. 

Mortality was the outcome for the analyses. Kaplan–Meier survival curves and log-rank test statistics were used to estimate the time to death (from the first interview) stratified by levels of fruit and vegetable consumption. Participants who were alive at the end of the observational period (31 October 2018) were censored. 

We conducted unadjusted and adjusted Cox proportional hazards regression models to explore the association between fruit and vegetable consumption and risk of all-cause mortality. The adjusted model included levels of fruit and vegetable consumption (with “low” level as the reference category) plus other potential confounders at baseline (gender, age, educational level, cohabiting, smoking status, level of physical activity, obesity, and number of chronic conditions). Interactions between levels of fruit and vegetable consumption and covariates were explored in the adjusted models. Hazard ratios (HRs) with their 95% confidence intervals (CI) were calculated.

The assumption of proportionality was explored by calculating plots of cumulative hazard functions across the independent variables. Violation in the assumption was not found. All analyses were performed using Stata version 13 for Windows (SE version 13, StataCorp: College Station, TX, USA) taking into account complex sampling design. Weights were used to adjust for differential probabilities of selection within households, and post-stratification corrections to the weights were made to match the samples to the socio-demographic distributions of the Spanish population. Statistical significance was set at *p* < 0.05.

### 3.5. Results

The mean age of the total sample was 74.8 years (95% CI = 74.49–75.12) ranging from 65 to 104, with 54.8% females, 54.7% cohabiting with someone, and 46.3% reporting low levels of education (Table 1). Some 37.2% reported being smokers or ex-smokers, 33.4% presented obesity, and 33.2% had low levels of physical activity. A total of 56.2% reported having two or more chronic conditions. Fruit and vegetable servings per day ranged from 0 to 18, with a mean of four servings (95% CI = 3.82–4.19). Participants with lower consumption (equal to or less than three servings per day) were more likely to be men, smokers, not frequently engaging in physical activity, frequent drinkers, and have a low educational level. 

**Figure 1:**
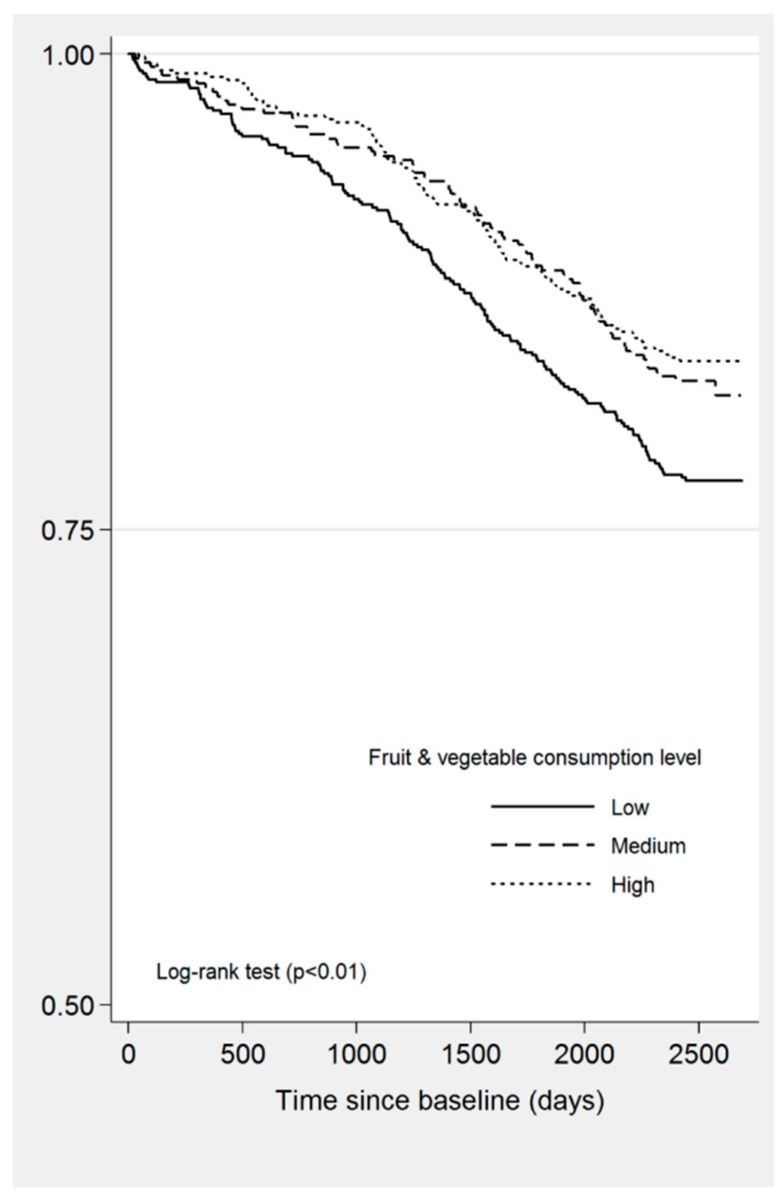
Kaplan–Meier estimated curves for cumulative survival by levels of fruit and vegetable consumption.

The minimum number of days of survival was 19 and the maximum 2688, with a mean of 2323.94 days (*SD* = 553.7). We observed 322 confirmed deceased cases (132 women and 190 men). The Kaplan–Meier estimated curves (Figure 1) showed that the level of fruit and vegetable consumption had a significant negative effect on survival. In the adjusted Cox proportional hazards regression model, only the interaction term fruit and vegetable consumption^*^ number of chronic conditions was found to be significant (*p* = 0.035). Table 2 presents the unadjusted and adjusted HRs and 95%CI. In the unadjusted model, both medium (HR = 1.68, 95%CI = 1.3–2.18) and high fruit (HR = 1.12, 95%CI = 1.1–1.14) consumption were significantly associated with higher risk of death. Other significant predictors of higher risk of mortality were being male, low levels of physical activity (compared with high), lower levels of education, being a smoker (current or past), and having three or more chronic conditions. The adjusted model including the interaction term between fruit and vegetable consumption and number of chronic conditions is presented in Table 2. In order to interpret the effect of fruit and vegetable consumption on time to death in the presence of interaction, HRs were calculated according to the number of chronic conditions (Table 3). The proportion of deceased people was significantly higher (*p* < 0.001) among those who had three or more CCs (*n* = 112, 25.8%) compared to those with none or one CC (*n* = 127, 16.3%) or two CCs (*n* = 83, 15.5%). Subjects who consumed five or more servings of fruit and vegetable per day and had two chronic conditions were at 27% less risk of mortality (HR = 0.38, 95%CI = 0.21–0.69, *p* = 0.002) compared with participants consuming three or fewer servings, while other covariates held constant. However, fruit and vegetable consumption had no impact on time to death among subjects who reported none or one CC, or three or more. 

Comparison between “medium” and “high” levels of fruit and vegetable consumption; (1) in the none or one CC group: *p* = 0.66; (2) in the two CC: *p* = 0.13; (3) and in the three or more CC: *p* = 0.596

**Figure 2:**
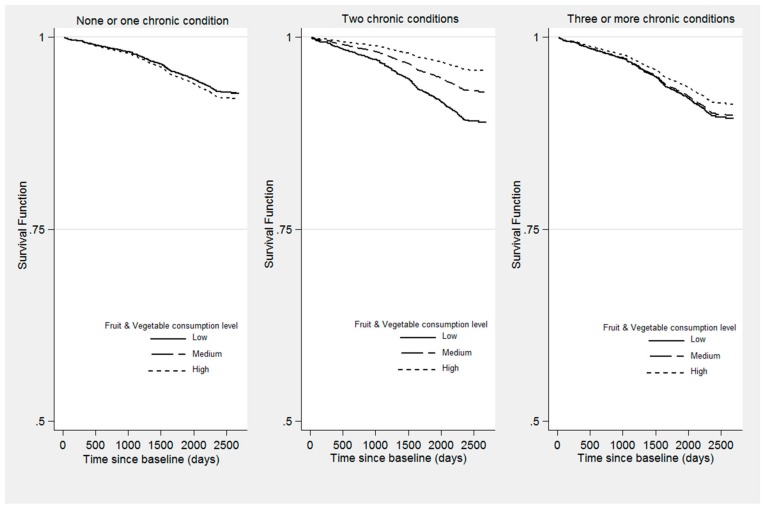
Survival function according to the level of fruit and vegetable consumption and number of chronic conditions (*n* = 1699). The first subfigure (left) shows the survival curves according to the three levels of fruit and vegetable intake for people with none or one chronic condition, the second (middle) for those with two chronic conditions and the third (right) shows survival curves associated with fruit and vegetable consumption for respondents with three or more chronic conditions. Note: Survival functions calculated from the adjusted Cox proportional hazards model presented in Table 2. All covariates were set equal to zero.

Survival curves as a function of levels of fruit and vegetable consumption and number of chronic conditions are displayed in Figure 2 for a specific pattern of covariates (i.e., females, not cohabiting, never smoked, no education/primary school studies, non-obese, lifetime abstainers, high level of physical activity, and age 74.8 years old). For people with two chronic conditions, the probability of surviving until the end of the study was significantly greater if they consumed five or more servings per day of fruit and vegetables, compared with those who ate three or fewer. 

## 4. Discussion

This study sought to determine the effect of fruit and vegetable consumption on all-cause mortality in a representative sample of Spanish community-dwelling older adults who were followed up for a period of approximately 6 years. Our results show that consuming five or more servings per day increases the probability of surviving in the general older population with two chronic conditions by 27%, compared to those who consume three or fewer servings per day. However, this beneficial effect of fruit and vegetable consumption is not found among participants with none or one chronic condition, or three or more. 

Most participants (65.7%) in the study did not adhere to the WHO recommendation of consuming a minimum of five servings of fruit and vegetables per day, with the median of servings per day being four. In a very large Spanish sample of university graduates, the mean consumption of fruits and vegetables was 343 g/day and 525 g/day [36], respectively, with an equivalence of approximately four and six portions of 80 g. However, few epidemiological studies have described the patterns of fruit and vegetable consumption among the older Spanish population. For example, in the Seniors-ENRICA study, a population-based cohort of Spanish older adults aged 60+, a total of 22.5% participants reported having five or more portions of fruit and vegetables a day [23], slightly inferior to the 34.3% found in our study. These discrepancies could be explained by the different tools used to assess fruit and vegetable consumption. 

Previous population-based studies have repeatedly reported an inverse association between fruit and vegetable consumption, and all-cause and disease-specific mortality in the older population [37,38,39], although some studies have reported inconsistent findings about whether this greater risk of all-cause mortality might be mainly due to CVD-related or non-cardiovascular-related deaths, such as cancer [40]. Our findings contribute to this evidence by showing that this effect is exerted through a protective effect in the presence of multimorbidity. Additionally, the consumption of both fruit and vegetables rather than the consumption of only fruit or vegetables seems to be especially beneficial for reducing the risk of CVD [39] and non-cardiovascular diseases [3]. Our findings also support the recommendation of a minimum of five servings per day of fruit and vegetables whereas there were no differences in terms of increased risk of mortality among older adults with low (equal to or less than three servings/d) or medium (four servings/d) consumption. This is in line with previous studies that investigated the risk of all-cause mortality associated with a dose-response of fruit and vegetable consumption in a large population-based cohort aged 45–83 [8]. The authors found that consuming fewer than five servings a day was associated with progressively shorter survival and higher mortality rate, whereas consuming more than five servings did not add any benefits with respect to survival. 

A systematic review conducted by Nunes et al [41] showed an overall positive association between multimorbidity (defined as the presence of two or more chronic diseases) and mortality (HR = 1.44, 95%CI = 134–1.55). In the unadjusted model, we found that only three or more chronic conditions were related to a 62% higher probability of having a shorter survival and dying, compared to those with none or one chronic condition. We found that the beneficial effect of consuming five or more fruit and vegetable servings per day is exerted in those having two CCs, but not three or more. Additionally, this protective effect seems to be beyond the confounding effects of other risk factors, such as obesity, physical activity, smoking, gender, or educational level. Participants suffering from three or more chronic conditions might represent complex patients, who might be in need of intensive care. The presence of multiple diseases is related to interactions between morbidities, inadequate use of medication, polypharmacy [42], and frailty [43]. Thus, the protective effect of high intake of fruit and vegetables might not be sufficient to reduce the risk of death in people with three or more CCs. It is also possible that older adults with three or more CCs have been given a prescription of a balanced diet, or have been advised to quit or reduce smoking and alcohol intake [44], which might in turn explain the lack of association between fruit and vegetable intake and time to death in this particular subgroup. Despite this, the beneficial effect of consuming five or more servings per day of fruit and vegetables could be huge. Taking into account that an important proportion of Spanish older adults do not reach the recommended five servings per day of fruit and vegetables along with the high prevalence of multimorbidity in this population, interventions promoting fruit and vegetable consumption among older adults might have a positive impact on reducing the risk of death and increasing their quality of life. Future research is needed to learn whether fruit and vegetable intake is particularly beneficial in reducing the risk of death for a particular pair of diseases. 

There are several mechanisms by which fruit and vegetable consumption can reduce the risk of mortality in older adults. Fruit and vegetables contain a variety of nutrients and phytochemicals (i.e., fibre, vitamin C, carotenoids, antioxidants, potassium, and flavonoids) that act through several biological mechanisms to reduce the risk of chronic conditions and premature mortality [3]. Greater intake of fruit and vegetables has also been linked to a greater adherence to the Mediterranean diet in older adults (characterized by abundant consumption of olive oil, minimally processed, locally grown vegetables, fruits, nuts, legumes, and cereals, and proteins coming mainly from fish and shellfish) [45] and to reduced consumption of sweet foods [46] which in turn might also prevent CVD [47], several types of cancer [48,49], cognitive decline, and dementia [50,51], while increasing longevity [52]. Our study did not include data on adherence to the Mediterranean diet or other potential dietary risk factors for non-communicable diseases and risk of mortality, such as consumption of red and processed meat [53] or ultraprocessed food [54]. More studies are needed to determine whether the beneficial effect of fruit and vegetable intake on the probability of survival among people with multimorbidity is maintained or attenuated by the presence of these diet-related risk factors. Additionally, the way in which fruits and vegetables are consumed (e.g., raw or cooked) might also play an important role in the potential protective factor among older adults with chronic conditions. Another mechanism by which fruit and vegetable consumption might impact the risk of mortality among older adults is the presence of unhealthy lifestyles among those who consume less fruit and vegetables. Previous research has indicated an inverse association between fruit and vegetable intake and smoking [55], alcohol consumption [56], obesity [57], and sedentarism [58]. The beneficial effects of consuming fruit and vegetables, such as lower systemic inflammation [59], reduced oxidative stress [60], and decreased platelet aggregation [61], may partially reduce the effects of smoking and alcohol intake [55,56]. However, we did not find significant interactions between fruit and vegetable consumption and smoking status, alcohol consumption, obesity, or low levels of physical activity. Future research is needed to replicate these results. 

Our study had some limitations. First, health variables, such as fruit and vegetable consumption, tobacco and physical activity, were self-reported, thus potentially leading to measurement errors or misclassification. Additionally, recall bias might also be present. Second, it was assumed that the fruit and vegetable intake pattern was unchanged during the follow-up period. Third, it is possible that the beneficial effect of fruit and vegetable consumption is not observed among participants with none or one chronic condition because they are more likely to survive during the follow-up period. Thus, longer periods of follow-up might be needed. Fourth, measuring fruit and vegetable consumption might be problematic. For example, the study did not extensively measure the dietary habits of the sample through a 24-hour dietary recall or a frequency questionnaire; thus, some measurement bias might have been introduced. Questions concerning the number of fruit and vegetable servings were asked once, yet they may be prone to seasonable bias as well. Additionally, these questions were aggregated, and the effect of this variable could be due to specific sorts of fruits and vegetables. Fourth, residual confounding might explain our findings. For example, consuming vitamin supplements or specific diet patterns such as the Mediterranean diet could be related to both fruit and vegetable consumption and mortality. However, findings were adjusted for several potential confounders, such as smoking status, alcohol consumption, physical activity, and obesity.

In sum, the finding that a high level of fruit and vegetable consumption (reaching the threshold of five or more servings per day) significantly reduces the risk of mortality among older adults with two chronic conditions has several implications. As has been shown in the present study, fruit and vegetable intake in the general population of older adults does not approach recommended levels. Interventions to increase fruit and vegetable intake in older adults should take into account their unique nutritional needs and barriers, as well as several characteristics that might influence their fruit and vegetable intake, such as appetite loss, tooth loss and oral problems, changes in perception of hunger, taste acuity and sense of smell (sometimes associated with drugs’ side effects), and mobility difficulties in shopping [18]. These factors should be taken into account when designing interventions to promote fruit and vegetable consumption geared to the older population. 

## Figures and Tables

**Table 1:** Baseline characteristics of the sample and comparison between levels of fruit and vegetable consumption.

Fruit & Vegetable Consumption					
	Total Sample (*n* = 1699)	Low (*n* = 669, 39.6%)	Medium (*n* = 448, 26.1%)	High (*n* = 582, 34.3%)	*p* Value
Death, *n (%)*	322 (18.6)	150 (21.9)	78 (18.7)	94 (14.7)	0.019
Age, *mean (95%CI)*	74.80 (74.49–75.12)	74.98 (74.46–75.5)	75.06 (74.43–75.68)	74.4 (73.57–75.24)	0.476
Females, *n (%)*	956 (54.8)	341 (49.7)	266 (58.8)	349 (57.6)	0.013
Cohabiting, *n (%)*	935 (54.7)	376 (54.4)	254 (56.9)	305 (53.4)	0.653
Educational level, *n (%)*					0.023
No education/Primary school	804 (46.3)	343 (51.7)	216 (47.5)	245 (39)	-
Secondary school	492 (30.1)	181 (27)	125 (30.6)	186 (33.4)	-
High school/University	403 (23.6)	145 (21.3)	107 (22)	151 (27.6)	0.791
Ever smoked, *n (%)*	620 (37.2)	278 (41.8)	150 (32.5)	192 (35.5)	0.008
Obesity, *n (%)*	606 (33.4)	237 (33.8)	156 (34.5)	213 (32.1)	0.803
Alcohol consumption, *n (%)*					<0.001
Lifetime abstainer	618 (36.5)	218 (31.9)	167 (38.7)	233 (40.1)	-
Occasional drinker	524 (31)	197 (28.6)	138 (30.4)	189 (34.2)	-
Frequent drinker	557 (32.5)	254 (39.5)	143 (30.9)	160 (25.7)	-
Number CC, *n (%)*					0.369
None or one	780 (43.8)	305 (43.9)	197 (42.2)	278 (44.9)	-
Two	482 (29.7)	188 (28.5)	130 (28.6)	164 (31.8)	-
Three or more	437 (26.5)	176 (27.6)	121 (29.2)	140 (23.3)	-
Level PA, *n (%)*					0.011
High	427 (25.2)	151 (21.6)	108 (21)	168 (32.7)	-
Medium	700 (41.6)	268 (40.6)	185 (45.1)	247 (40.1)	-
Low	572 (33.2)	250 (37.8)	155 (33.9)	167 (27.2)	-

Note: CC = Chronic conditions; PA = Physical activity; 95% CI = 95% Confidence interval; Unweighted frequencies, weighted proportions and means.

**Table 2:** Hazard ratios, Confidence intervals and *p* values for the unadjusted and adjusted Cox proportional hazards models (*n* = 1699).

	Unadjusted		Adjusted ^a^	
	HR (95% CI)	*p* Value	HR (95% CI)	*p* Value
*Fruit & veg consumption*				
Low (ref.)	-	-	-	-
Medium	0.83 (0.61–1.13)	0.802	1.0 (0.62–1.60)	0.989
High	**1.68 (1.3–2.18)**	**<0.001**	1.11 (0.70–1.74)	0.659
*Age*	**1.12 (1.1–1.14)**	**<0.001**	**1.13 (1.1–15)**	**<0.001**
*Gender*				
Female (ref.)	-	-	-	-
Male	**1.96 (1.49–2.57)**	**<0.001**	**3.31 (2.21–4.99)**	**<0.001**
*Marital status*				
Not cohabiting (ref.)	-	-	-	-
Cohabiting	0.84 (0.66–1.1)	0.169	0.9 (0.66–1.24)	0.523
*Educational level*				
No education/Primary school	-	-	-	-
Secondary school	**0.57 (0.39–0.83)**	**0.004**	0.77 (0.54–1.11)	0.168
High school/University	**0.67 (0.49–0.92)**	**0.013**	1.0 (0.74–1.35)	0.997
*Level PA*				
High (ref.)	-	-	-	-
Medium	1.45 (0.99–2.13)	0.056	1.28 (0.84–1.93)	0.246
Low	**2.3 (1.59–3.32)**	**<0.001**	**1.61 (1.07–2.43)**	**0.021**
*Smoking status*		-	-	-
Never smoked	-	-	-	-
Ever smoked	**1.38 (1.07–1.78)**	**0.013**	1.01 (0.73–1.39)	0.973
*Alcohol consumption*				
Lifetime abstainer (ref.)	-	-	-	-
Occasional drinker	1.13 (0.84–1.5)	0.414	0.97 (0.69–1.36)	0.87
Frequent drinker	0.93 (0.69–1.24)	0.625	**0.64 (0.46–0.91)**	**0.012**
*Obesity*				
Non-obese (ref.)	-	-	-	-
Obese	1.14 (0.82–1.57)	0.43	1.11 (0.8–1.55)	0.515
*Number CC*				
None/one (ref.)	-	-	-	-
Two	0.95 (0.67–1.37)	0.802	1.56 (0.91–2.65)	0.102
Three or more	**1.68 (1.3–2.18)**	**<0.001**	1.47 (0.99–2.18)	0.056
*Fruit & veg × number CC*				
Medium/two CC	-	-	0.63 (0.31–1.28)	0.199
Medium/three+ CC	-	-	0.97 (0.47–2.0)	0.930
High/two CC	-	-	**0.34 (0.18–0.65)**	**0.001**
High/three+ CC	-	-	0.74 (0.39–1.42)	0.360

*Note:* HR = Hazard ratio; 95% CI = 95% Confidence interval; CC = Chronic conditions; PA = Physical activity. In bold, significant effect. ^a^ The adjusted model included all the variables and the interaction term simultaneously.

**Table 3:** Adjusted hazard ratios of the effect of fruit and vegetable consumption by number of chronic conditions on all-cause mortality. Cox regression model (*n* = 1,699).

	None or One CC				Two CC				Three or More CC			
Level of Fruit & Vegetable Consumption	HR	95%CI		*p* Value	HR	95%CI		*p* Value	HR	95%CI		*p* Value
Low (ref.)	-	-	-	-	-	-	-	-	-	-	-	-
Medium	1.0	0.62	1.6	0.989	0.63	0.33	0.19	0.151	0.96	0.54	0.73	0.905
High	1.11	0.7	1.74	0.659	**0.38**	**0.21**	**0.69**	**0.002**	0.82	0.5	1.35	0.428

Note: HR = Hazard ratio; 95%CI = 95% Confidence interval; CC = Chronic conditions. In bold, significant HR. Model included the fruit & vegetable consumption*number CC interaction term and the following variables: age, gender, educational level, cohabiting, ever smoked, obesity, alcohol consumption, and level of physical activity (see Table 2).
